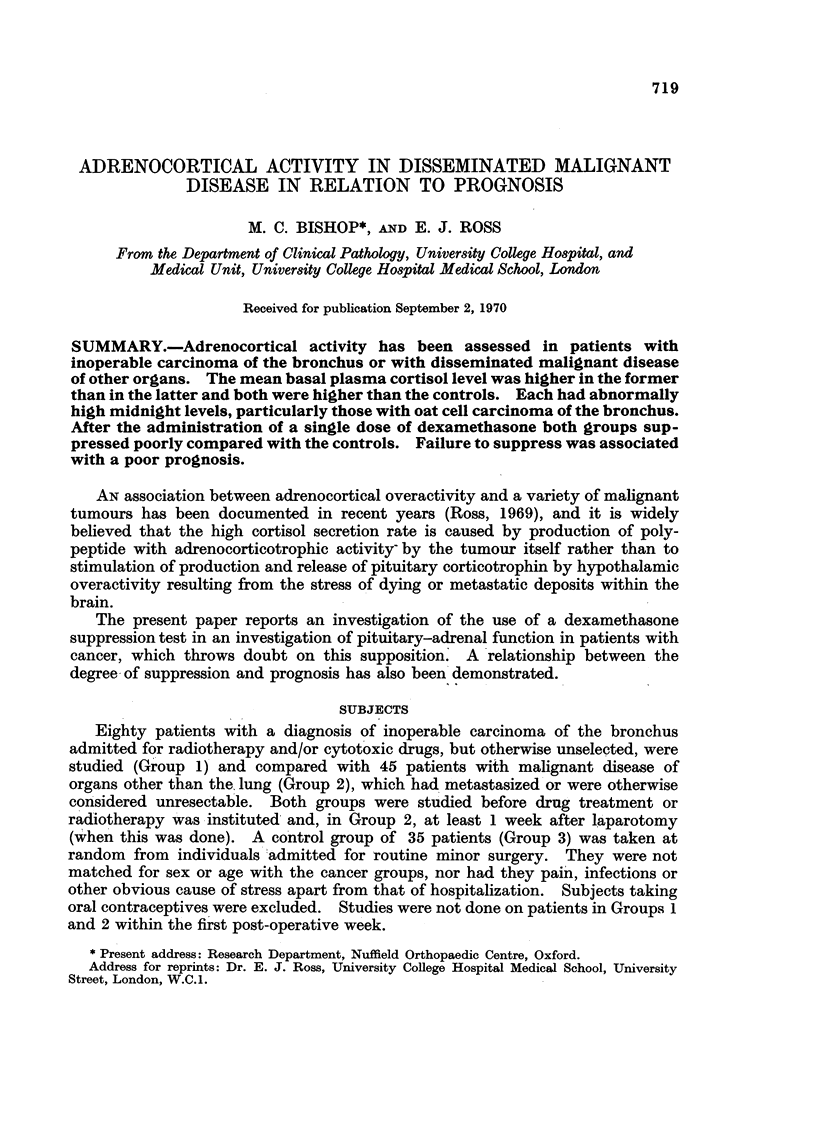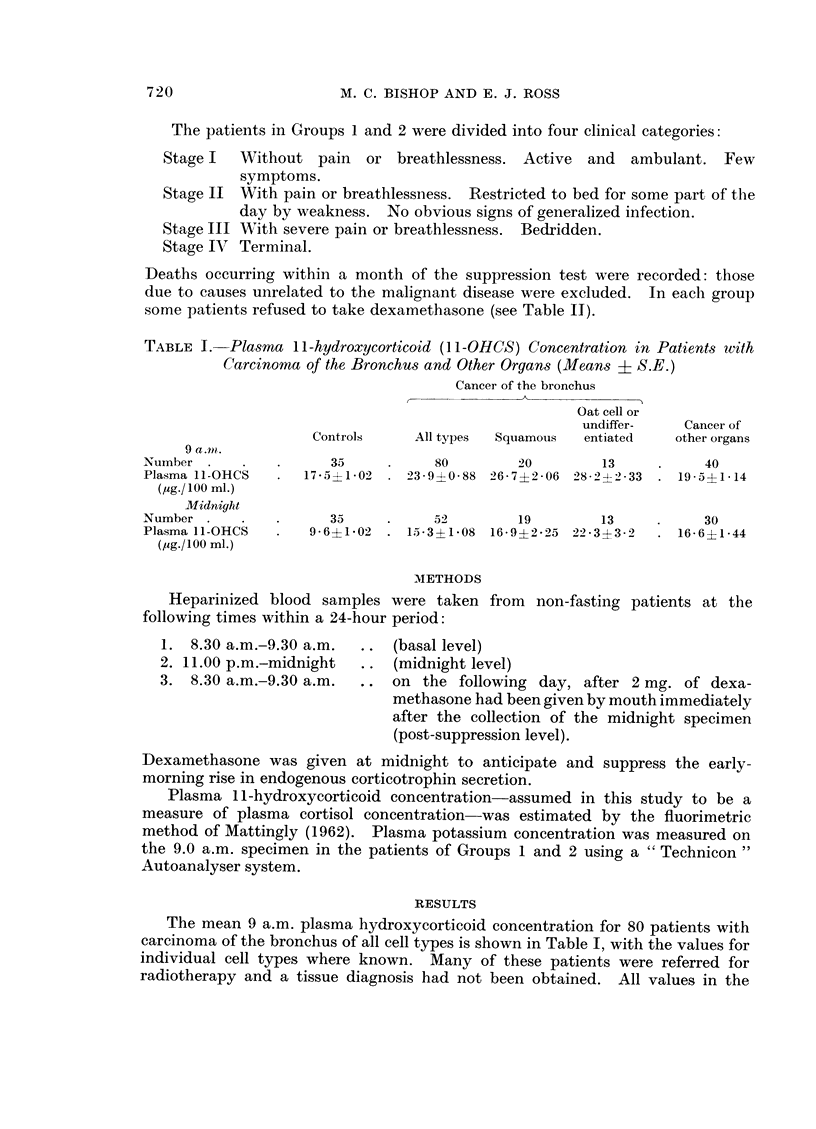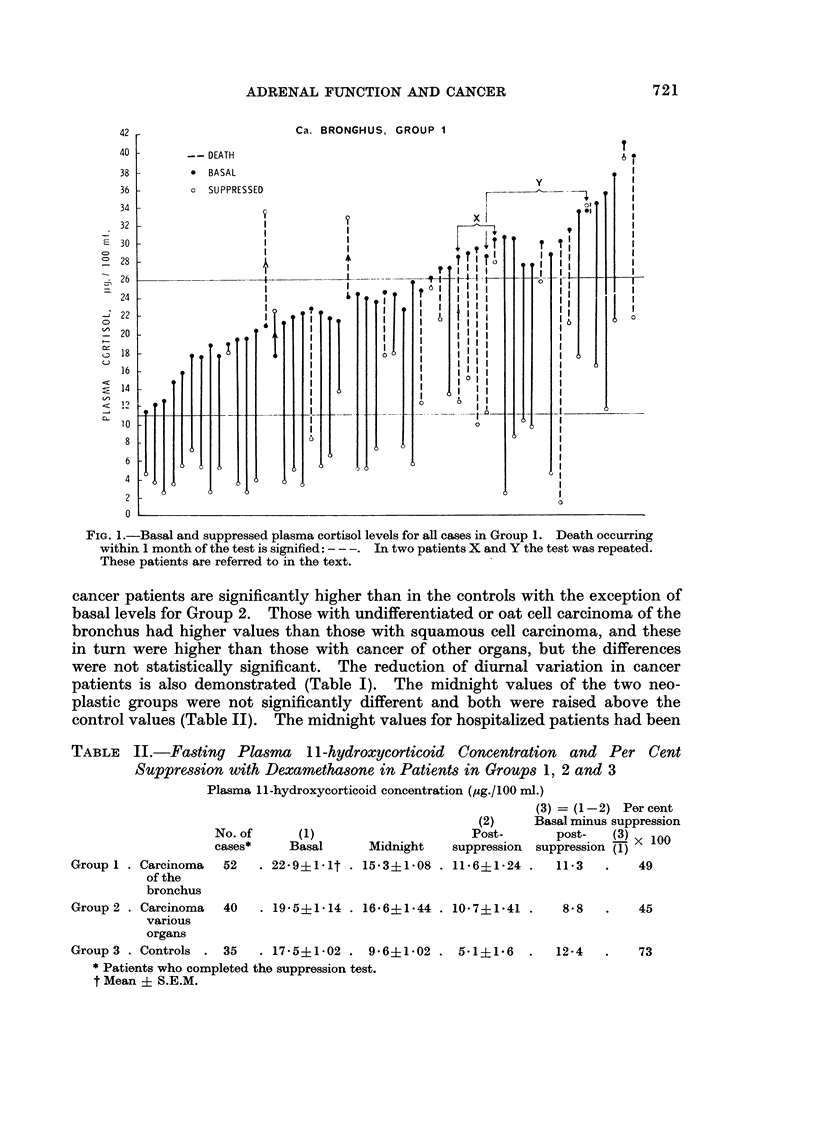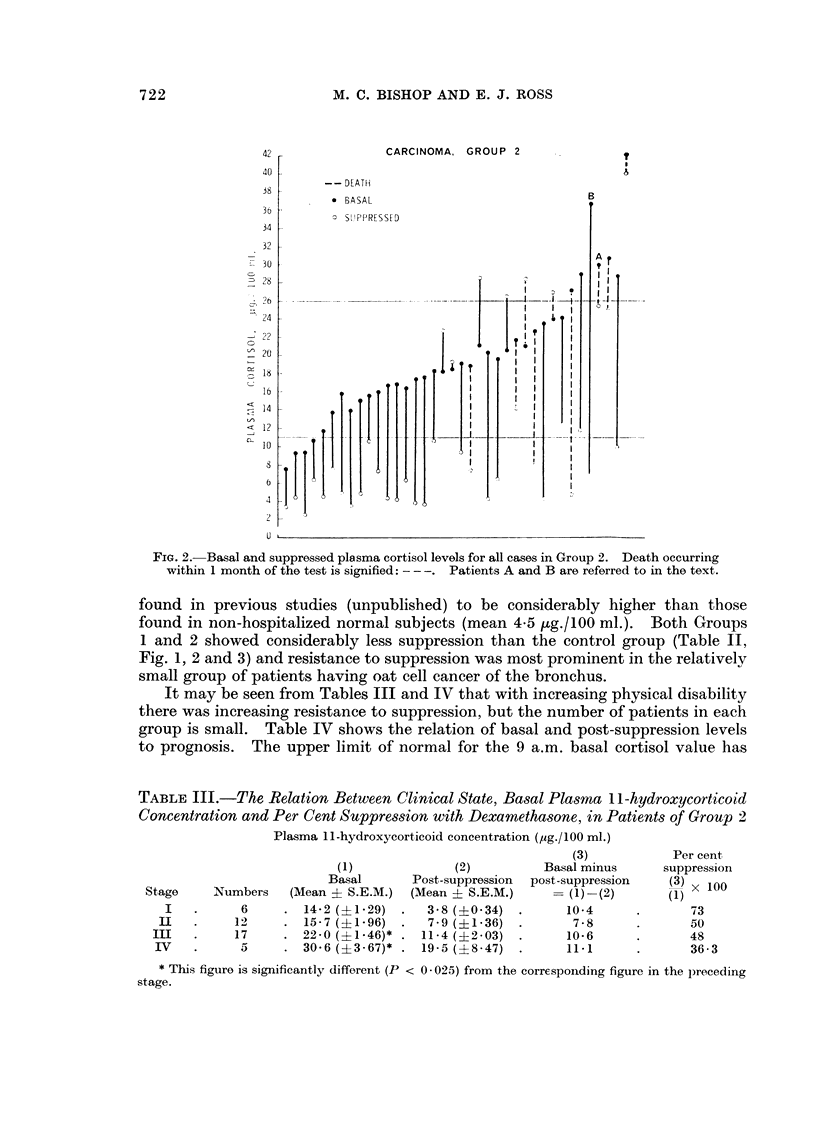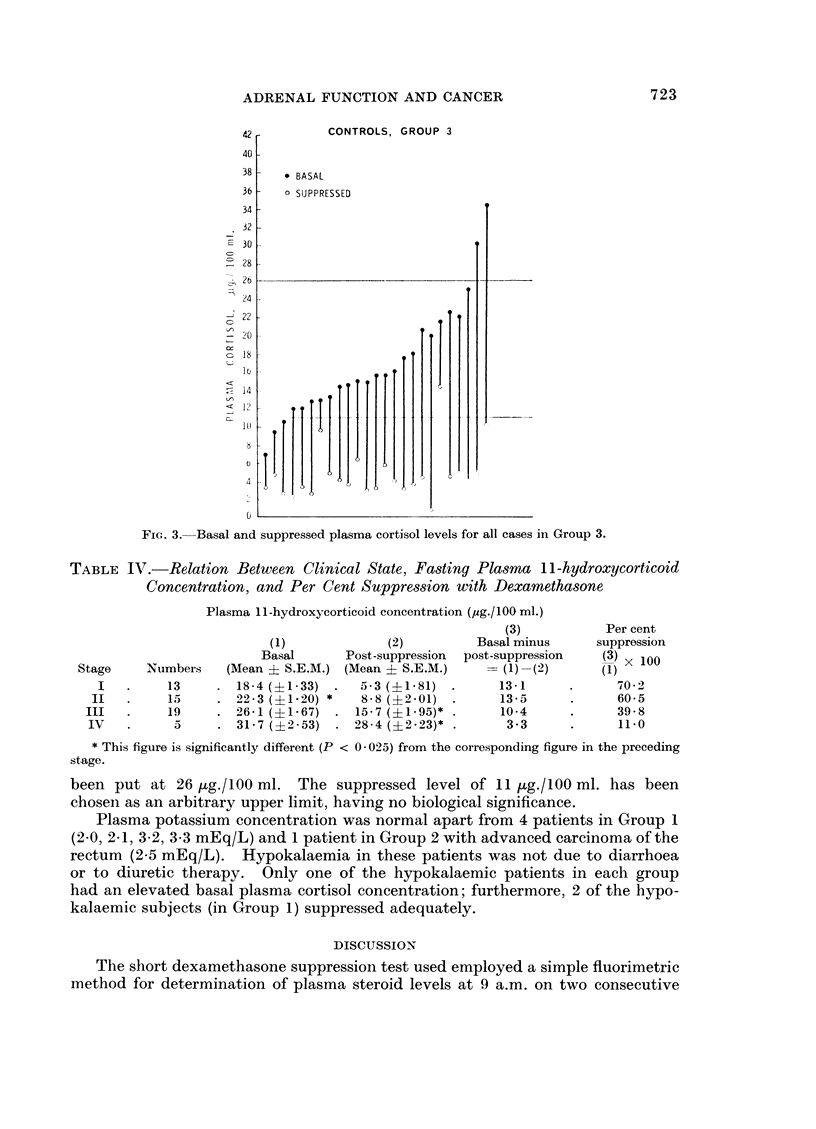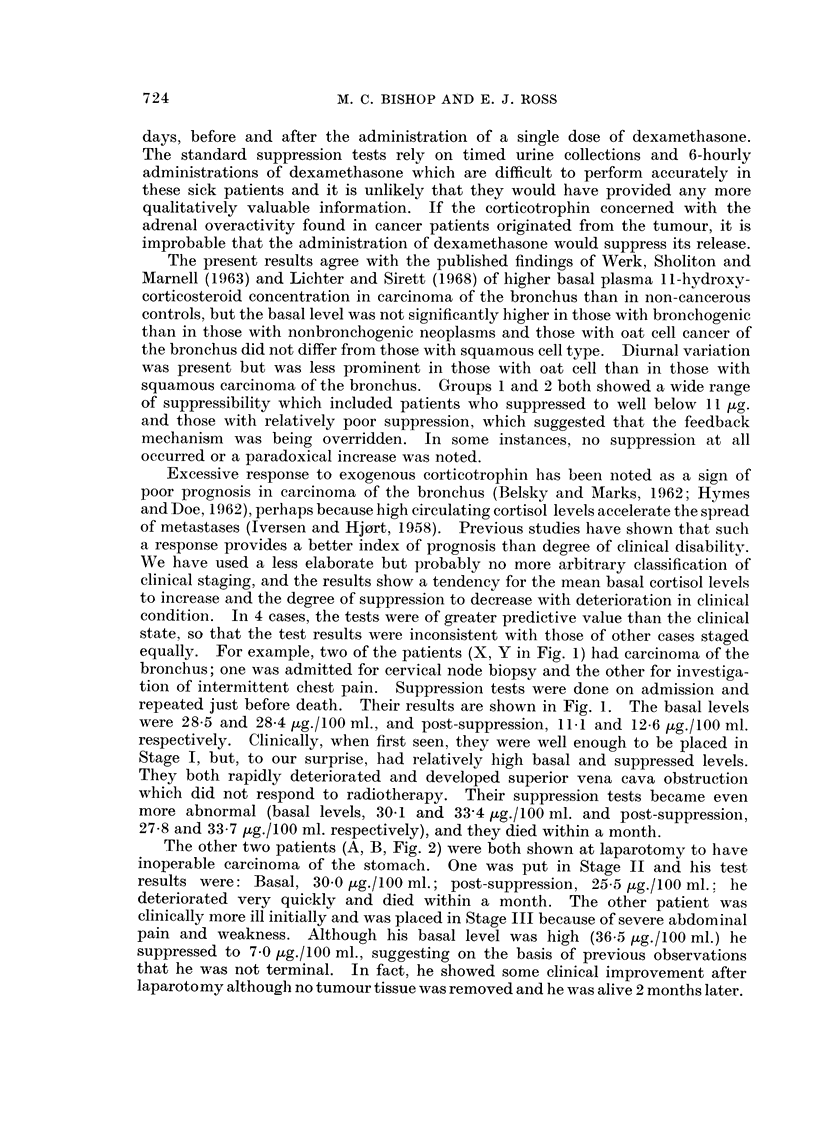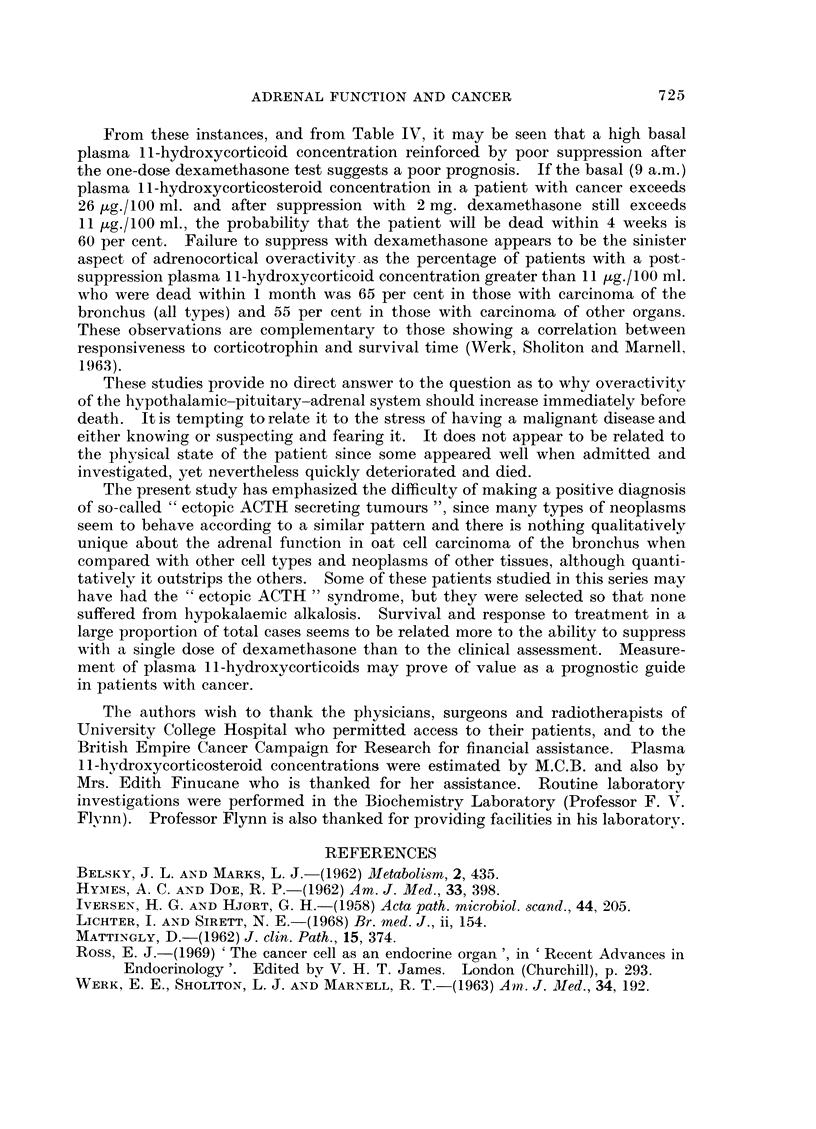# Adrenocortical Activity in Disseminated Malignant Disease in Relation to Prognosis

**DOI:** 10.1038/bjc.1970.86

**Published:** 1970-12

**Authors:** M. C. Bishop, E. J. Ross

## Abstract

Adrenocortical activity has been assessed in patients with inoperable carcinoma of the bronchus or with disseminated malignant disease of other organs. The mean basal plasma cortisol level was higher in the former than in the latter and both were higher than the controls. Each had abnormally high midnight levels, particularly those with oat cell carcinoma of the bronchus. After the administration of a single dose of dexamethasone both groups suppressed poorly compared with the controls. Failure to suppress was associated with a poor prognosis.


### ADRENOCORTICAL ACTIVITY IN DISSEMINATED MALIGNANT DISEASE IN RELATION TO PROGNOSIS

M. C. BISHOP*, AND E. J. ROSS

*From the Department of Clinical Pathology, University College Hospital, and Medical Unit, University College Hospital Medical School, London*

Received for publication September 2, 1970

**SUMMARY.—Adrenocortical activity has been assessed in patients with inoperable carcinoma of the bronchus or with disseminated malignant disease of other organs. The mean basal plasma cortisol level was higher in the former than in the latter and both were higher than the controls. Each had abnormally high midnight levels, particularly those with oat cell carcinoma of the bronchus. After the administration of a single dose of dexamethasone both groups suppressed poorly compared with the controls. Failure to suppress was associated with a poor prognosis.**

AN association between adrenocortical overactivity and a variety of malignant tumours has been documented in recent years (Ross, 1969), and it is widely believed that the high cortisol secretion rate is caused by production of polypeptide with adrenocorticotrophic activity by the tumour itself rather than to stimulation of production and release of pituitary corticotrophin by hypothalamic overactivity resulting from the stress of dying or metastatic deposits within the brain.

The present paper reports an investigation of the use of a dexamethasone suppression test in an investigation of pituitary–adrenal function in patients with cancer, which throws doubt on this supposition. A relationship between the degree of suppression and prognosis has also been demonstrated.

### SUBJECTS

Eighty patients with a diagnosis of inoperable carcinoma of the bronchus admitted for radiotherapy and/or cytotoxic drugs, but otherwise unselected, were studied (Group 1) and compared with 45 patients with malignant disease of organs other than the lung (Group 2), which had metastasized or were otherwise considered unresectable. Both groups were studied before drug treatment or radiotherapy was instituted and, in Group 2, at least 1 week after laparotomy (when this was done). A control group of 35 patients (Group 3) was taken at random from individuals admitted for routine minor surgery. They were not matched for sex or age with the cancer groups, nor had they pain, infections or other obvious cause of stress apart from that of hospitalization. Subjects taking oral contraceptives were excluded. Studies were not done on patients in Groups 1 and 2 within the first post-operative week.

* Present address: Research Department, Nuffield Orthopaedic Centre, Oxford.
Address for reprints: Dr. E. J. Ross, University College Hospital Medical School, University Street, London, W.C.1.

The patients in Groups 1 and 2 were divided into four clinical categories:

| | |
|---|---|
| Stage I | Without pain or breathlessness. Active and ambulant. Few symptoms. |
| Stage II | With pain or breathlessness. Restricted to bed for some part of the day by weakness. No obvious signs of generalized infection. |
| Stage III | With severe pain or breathlessness. Bedridden. |
| Stage IV | Terminal. |

Deaths occurring within a month of the suppression test were recorded: those due to causes unrelated to the malignant disease were excluded. In each group some patients refused to take dexamethasone (see Table II).

TABLE I.—*Plasma 11-hydroxycorticoid (11-OHCS) Concentration in Patients with Carcinoma of the Bronchus and Other Organs (Means ± S.E.)*

| | Controls | Cancer of the bronchus: All types | Cancer of the bronchus: Squamous | Cancer of the bronchus: Oat cell or undifferentiated | Cancer of other organs |
|---|---|---|---|---|---|
| *9 a.m.* | | | | | |
| Number | 35 | 80 | 20 | 13 | 40 |
| Plasma 11-OHCS (μg./100 ml.) | 17·5±1·02 | 23·9±0·88 | 26·7±2·06 | 28·2±2·33 | 19·5±1·14 |
| *Midnight* | | | | | |
| Number | 35 | 52 | 19 | 13 | 30 |
| Plasma 11-OHCS (μg./100 ml.) | 9·6±1·02 | 15·3±1·08 | 16·9±2·25 | 22·3±3·2 | 16·6±1·44 |

### METHODS

Heparinized blood samples were taken from non-fasting patients at the following times within a 24-hour period:

1. 8.30 a.m.–9.30 a.m. .. (basal level)
2. 11.00 p.m.–midnight .. (midnight level)
3. 8.30 a.m.–9.30 a.m. .. on the following day, after 2 mg. of dexamethasone had been given by mouth immediately after the collection of the midnight specimen (post-suppression level).

Dexamethasone was given at midnight to anticipate and suppress the early-morning rise in endogenous corticotrophin secretion.

Plasma 11-hydroxycorticoid concentration—assumed in this study to be a measure of plasma cortisol concentration—was estimated by the fluorimetric method of Mattingly (1962). Plasma potassium concentration was measured on the 9.0 a.m. specimen in the patients of Groups 1 and 2 using a " Technicon " Autoanalyser system.

### RESULTS

The mean 9 a.m. plasma hydroxycorticoid concentration for 80 patients with carcinoma of the bronchus of all cell types is shown in Table I, with the values for individual cell types where known. Many of these patients were referred for radiotherapy and a tissue diagnosis had not been obtained. All values in the

FIG. 1.—Basal and suppressed plasma cortisol levels for all cases in Group 1. Death occurring within 1 month of the test is signified: – – –. In two patients X and Y the test was repeated. These patients are referred to in the text.

cancer patients are significantly higher than in the controls with the exception of basal levels for Group 2. Those with undifferentiated or oat cell carcinoma of the bronchus had higher values than those with squamous cell carcinoma, and these in turn were higher than those with cancer of other organs, but the differences were not statistically significant. The reduction of diurnal variation in cancer patients is also demonstrated (Table I). The midnight values of the two neoplastic groups were not significantly different and both were raised above the control values (Table II). The midnight values for hospitalized patients had been

TABLE II.—*Fasting Plasma* 11-*hydroxycorticoid Concentration and Per Cent Suppression with Dexamethasone in Patients in Groups* 1, 2 *and* 3

Plasma 11-hydroxycorticoid concentration (µg./100 ml.)

| | | No. of cases* | (1) Basal | Midnight | (2) Post-suppression | (3) = (1 − 2) Basal minus post-suppression | Per cent suppression $\frac{(3)}{(1)} \times 100$ |
|---|---|---|---|---|---|---|---|
| Group 1 | Carcinoma of the bronchus | 52 | 22·9±1·1† | 15·3±1·08 | 11·6±1·24 | 11·3 | 49 |
| Group 2 | Carcinoma various organs | 40 | 19·5±1·14 | 16·6±1·44 | 10·7±1·41 | 8·8 | 45 |
| Group 3 | Controls | 35 | 17·5±1·02 | 9·6±1·02 | 5·1±1·6 | 12·4 | 73 |

* Patients who completed the suppression test.
† Mean ± S.E.M.

FIG. 2.—Basal and suppressed plasma cortisol levels for all cases in Group 2. Death occurring within 1 month of the test is signified: – – –. Patients A and B are referred to in the text.

found in previous studies (unpublished) to be considerably higher than those found in non-hospitalized normal subjects (mean 4·5 µg./100 ml.). Both Groups 1 and 2 showed considerably less suppression than the control group (Table II, Fig. 1, 2 and 3) and resistance to suppression was most prominent in the relatively small group of patients having oat cell cancer of the bronchus.

It may be seen from Tables III and IV that with increasing physical disability there was increasing resistance to suppression, but the number of patients in each group is small. Table IV shows the relation of basal and post-suppression levels to prognosis. The upper limit of normal for the 9 a.m. basal cortisol value has

TABLE III.—*The Relation Between Clinical State, Basal Plasma* 11-*hydroxycorticoid Concentration and Per Cent Suppression with Dexamethasone, in Patients of Group* 2

| | | Plasma 11-hydroxycorticoid concentration (µg./100 ml.) | | | |
|---|---|---|---|---|---|
| Stage | Numbers | (1) Basal (Mean ± S.E.M.) | (2) Post-suppression (Mean ± S.E.M.) | (3) Basal minus post-suppression = (1)−(2) | Per cent suppression $\frac{(3)}{(1)} \times 100$ |
| I | 6 | 14·2 (±1·29) | 3·8 (±0·34) | 10·4 | 73 |
| II | 12 | 15·7 (±1·96) | 7·9 (±1·36) | 7·8 | 50 |
| III | 17 | 22·0 (±1·46)* | 11·4 (±2·03) | 10·6 | 48 |
| IV | 5 | 30·6 (±3·67)* | 19·5 (±8·47) | 11·1 | 36·3 |

* This figure is significantly different ($P < 0·025$) from the corresponding figure in the preceding stage.

FIG. 3.—Basal and suppressed plasma cortisol levels for all cases in Group 3.

TABLE IV.—*Relation Between Clinical State, Fasting Plasma 11-hydroxycorticoid Concentration, and Per Cent Suppression with Dexamethasone*

| | | Plasma 11-hydroxycorticoid concentration (µg./100 ml.) | | | |
|---|---|---|---|---|---|
| Stage | Numbers | (1) Basal (Mean ± S.E.M.) | (2) Post-suppression (Mean ± S.E.M.) | (3) Basal minus post-suppression = (1)−(2) | Per cent suppression $\frac{(3)}{(1)} \times 100$ |
| I | 13 | 18·4 (±1·33) | 5·3 (±1·81) | 13·1 | 70·2 |
| II | 15 | 22·3 (±1·20) * | 8·8 (±2·01) | 13·5 | 60·5 |
| III | 19 | 26·1 (±1·67) | 15·7 (±1·95)* | 10·4 | 39·8 |
| IV | 5 | 31·7 (±2·53) | 28·4 (±2·23)* | 3·3 | 11·0 |

* This figure is significantly different ($P < 0·025$) from the corresponding figure in the preceding stage.

been put at 26 µg./100 ml. The suppressed level of 11 µg./100 ml. has been chosen as an arbitrary upper limit, having no biological significance.

Plasma potassium concentration was normal apart from 4 patients in Group 1 (2·0, 2·1, 3·2, 3·3 mEq/L) and 1 patient in Group 2 with advanced carcinoma of the rectum (2·5 mEq/L). Hypokalaemia in these patients was not due to diarrhoea or to diuretic therapy. Only one of the hypokalaemic patients in each group had an elevated basal plasma cortisol concentration; furthermore, 2 of the hypokalaemic subjects (in Group 1) suppressed adequately.

### DISCUSSION

The short dexamethasone suppression test used employed a simple fluorimetric method for determination of plasma steroid levels at 9 a.m. on two consecutive days, before and after the administration of a single dose of dexamethasone. The standard suppression tests rely on timed urine collections and 6-hourly administrations of dexamethasone which are difficult to perform accurately in these sick patients and it is unlikely that they would have provided any more qualitatively valuable information. If the corticotrophin concerned with the adrenal overactivity found in cancer patients originated from the tumour, it is improbable that the administration of dexamethasone would suppress its release.

The present results agree with the published findings of Werk, Sholiton and Marnell (1963) and Lichter and Sirett (1968) of higher basal plasma 11-hydroxy-corticosteroid concentration in carcinoma of the bronchus than in non-cancerous controls, but the basal level was not significantly higher in those with bronchogenic than in those with nonbronchogenic neoplasms and those with oat cell cancer of the bronchus did not differ from those with squamous cell type. Diurnal variation was present but was less prominent in those with oat cell than in those with squamous carcinoma of the bronchus. Groups 1 and 2 both showed a wide range of suppressibility which included patients who suppressed to well below 11 $\mu$g. and those with relatively poor suppression, which suggested that the feedback mechanism was being overridden. In some instances, no suppression at all occurred or a paradoxical increase was noted.

Excessive response to exogenous corticotrophin has been noted as a sign of poor prognosis in carcinoma of the bronchus (Belsky and Marks, 1962; Hymes and Doe, 1962), perhaps because high circulating cortisol levels accelerate the spread of metastases (Iversen and Hjørt, 1958). Previous studies have shown that such a response provides a better index of prognosis than degree of clinical disability. We have used a less elaborate but probably no more arbitrary classification of clinical staging, and the results show a tendency for the mean basal cortisol levels to increase and the degree of suppression to decrease with deterioration in clinical condition. In 4 cases, the tests were of greater predictive value than the clinical state, so that the test results were inconsistent with those of other cases staged equally. For example, two of the patients (X, Y in Fig. 1) had carcinoma of the bronchus; one was admitted for cervical node biopsy and the other for investigation of intermittent chest pain. Suppression tests were done on admission and repeated just before death. Their results are shown in Fig. 1. The basal levels were 28·5 and 28·4 $\mu$g./100 ml., and post-suppression, 11·1 and 12·6 $\mu$g./100 ml. respectively. Clinically, when first seen, they were well enough to be placed in Stage I, but, to our surprise, had relatively high basal and suppressed levels. They both rapidly deteriorated and developed superior vena cava obstruction which did not respond to radiotherapy. Their suppression tests became even more abnormal (basal levels, 30·1 and 33·4 $\mu$g./100 ml. and post-suppression, 27·8 and 33·7 $\mu$g./100 ml. respectively), and they died within a month.

The other two patients (A, B, Fig. 2) were both shown at laparotomy to have inoperable carcinoma of the stomach. One was put in Stage II and his test results were: Basal, 30·0 $\mu$g./100 ml.; post-suppression, 25·5 $\mu$g./100 ml.; he deteriorated very quickly and died within a month. The other patient was clinically more ill initially and was placed in Stage III because of severe abdominal pain and weakness. Although his basal level was high (36·5 $\mu$g./100 ml.) he suppressed to 7·0 $\mu$g./100 ml., suggesting on the basis of previous observations that he was not terminal. In fact, he showed some clinical improvement after laparotomy although no tumour tissue was removed and he was alive 2 months later.

From these instances, and from Table IV, it may be seen that a high basal plasma 11-hydroxycorticoid concentration reinforced by poor suppression after the one-dose dexamethasone test suggests a poor prognosis. If the basal (9 a.m.) plasma 11-hydroxycorticosteroid concentration in a patient with cancer exceeds 26 $\mu$g./100 ml. and after suppression with 2 mg. dexamethasone still exceeds 11 $\mu$g./100 ml., the probability that the patient will be dead within 4 weeks is 60 per cent. Failure to suppress with dexamethasone appears to be the sinister aspect of adrenocortical overactivity as the percentage of patients with a post-suppression plasma 11-hydroxycorticoid concentration greater than 11 $\mu$g./100 ml. who were dead within 1 month was 65 per cent in those with carcinoma of the bronchus (all types) and 55 per cent in those with carcinoma of other organs. These observations are complementary to those showing a correlation between responsiveness to corticotrophin and survival time (Werk, Sholiton and Marnell, 1963).

These studies provide no direct answer to the question as to why overactivity of the hypothalamic–pituitary–adrenal system should increase immediately before death. It is tempting to relate it to the stress of having a malignant disease and either knowing or suspecting and fearing it. It does not appear to be related to the physical state of the patient since some appeared well when admitted and investigated, yet nevertheless quickly deteriorated and died.

The present study has emphasized the difficulty of making a positive diagnosis of so-called " ectopic ACTH secreting tumours ", since many types of neoplasms seem to behave according to a similar pattern and there is nothing qualitatively unique about the adrenal function in oat cell carcinoma of the bronchus when compared with other cell types and neoplasms of other tissues, although quantitatively it outstrips the others. Some of these patients studied in this series may have had the " ectopic ACTH " syndrome, but they were selected so that none suffered from hypokalaemic alkalosis. Survival and response to treatment in a large proportion of total cases seems to be related more to the ability to suppress with a single dose of dexamethasone than to the clinical assessment. Measurement of plasma 11-hydroxycorticoids may prove of value as a prognostic guide in patients with cancer.

The authors wish to thank the physicians, surgeons and radiotherapists of University College Hospital who permitted access to their patients, and to the British Empire Cancer Campaign for Research for financial assistance. Plasma 11-hydroxycorticosteroid concentrations were estimated by M.C.B. and also by Mrs. Edith Finucane who is thanked for her assistance. Routine laboratory investigations were performed in the Biochemistry Laboratory (Professor F. V. Flynn). Professor Flynn is also thanked for providing facilities in his laboratory.

### REFERENCES

Belsky, J. L. and Marks, L. J.—(1962) *Metabolism*, **2**, 435.
Hymes, A. C. and Doe, R. P.—(1962) *Am. J. Med.*, **33**, 398.
Iversen, H. G. and Hjørt, G. H.—(1958) *Acta path. microbiol. scand.*, **44**, 205.
Lichter, I. and Sirett, N. E.—(1968) *Br. med. J.*, ii, 154.
Mattingly, D.—(1962) *J. clin. Path.*, **15**, 374.
Ross, E. J.—(1969) ' The cancer cell as an endocrine organ ', in ' Recent Advances in Endocrinology '. Edited by V. H. T. James. London (Churchill), p. 293.
Werk, E. E., Sholiton, L. J. and Marnell, R. T.—(1963) *Am. J. Med.*, **34**, 192.